# Implementing the Safer Baby Bundle for stillbirth prevention across Queensland maternity services using a modified breakthrough series collaborative

**DOI:** 10.1186/s43058-026-00921-2

**Published:** 2026-04-13

**Authors:** Michael Rice, Simin Arabshahi, Colette McIntyre, Adam Burns, Jocelyn Toohill, Kym Warhurst, Anne Bousfield, David Ellwood, Vicki Flenady, Christine Andrews

**Affiliations:** 1 Clinical Excellence Queensland, Patient Safety and Quality, 15 Butterfield Street, Herston, Brisbane, QLD 4006 Australia; 2 Clinical Excellence Queensland, Office of the Chief Nursing and Midwifery Officer, 15 Butterfield Street, Brisbane, QLD 4006 Australia; 3 Mater Misericordiae Ltd, Raymond Terrace, South Brisbane, QLD 4101 Australia; 4 Roma Hospital, South-West Hospital and Health Service, 197–243 McDowall Street, Roma, QLD 4455 Australia; 5 Centre of Research Excellence in Stillbirth, Raymond Terrace, Mater Research Institute-University of Queensland, Level 3 Aubigny Place, Brisbane, QLD 4101 Australia; 6 School of Medicine and Dentistry, Griffith University, Parklands Drive, Southport, Gold Coast, QLD 4215 Australia; 7 Gold Coast University Hospital, 1 Hospital Boulevard, Southport, Gold Coast, QLD 4215 Australia

**Keywords:** Quality improvement, Maternity, Stillbirth, Breakthrough series collaborative, Safer baby bundle

## Abstract

**Background:**

Australia’s ≥ 28‑weeks stillbirth rate is 19.5% higher than that of high‑income countries with the lowest rates. The Safer Baby Bundle (SBB) is a national initiative to reduce stillbirth in Australia targeting five components of antenatal care and is the key prevention strategy within National Stillbirth Action and Implementation Plan. This paper reports the experience in one Australian state that delivered the Safer Baby Bundle Improvement Project (SBBIP) to support clinical staff implementing the SBB. In the setting of extreme maternity workforce challenges compounded by the COVID-19 pandemic, the implementation used a modified Breakthrough Series Collaborative (BTS).

**Methods:**

Over an 18-month period, antenatal services across Queensland used a modified BTS Collaborative approach, removing the need for teams to document Plan-Do-Study-Act cycles, use statistical process control (SPC) charts, document project progress scores and monthly reporting. Engagement during the improvement effort was assessed. A before-and-after multimethod study was used to evaluate the program. Routinely collected perinatal data, clinical audits, project administrative data and surveys of healthcare professionals and women receiving antenatal care were used to measure improvements before and after implementation, and logistic regression interrupted time series (ITS) analyses were used for comparisons of the outcomes.

**Results:**

Despite disruptions from the COVID-19 pandemic, the SBB was implemented across antenatal services, and the modified BTS implementation strategy achieved positive results. Eighty-nine percent of the 45 enrolled teams were actively engaged in the improvement effort across the SBBIP and all (100%) implemented one or more change ideas. Post implementation, improvements were observed in all key process measures and balance measures (planned singleton birth before 39 weeks, late preterm and early-term singleton births), whereas other measures remained unchanged. The stillbirth rate ≥ 28 weeks in singletons remained at 2.1 per 1000 births before and after implementation. ITS analyses of eligible measures supported these patterns.

**Conclusions:**

In an environment challenged by workforce shortages, high workload demand, and competing priorities (global pandemic), a modified BTS Collaborative approach is a useful model to implement improvement at scale to reduce stillbirth risk factors.

**Supplementary Information:**

The online version contains supplementary material available at 10.1186/s43058-026-00921-2.

Contributions to the literature

- The IHI’s breakthrough series collaborative methodology contains prescriptive elements proven to achieve improvement at scale when closely followed; however, it is not clear from the literature which elements carry the most influence toward achieving a stated aim.
- In an environment challenged by resource and time constraints, we explored whether improvement at scale could be achieved after removing four BTS elements.
- The findings in this paper suggest that improvement is possible at scale in the absence of these elements, which addresses a gap in the literature around which elements may be less impactful in achieving a stated aim.

## Introduction

Australia lags behind other high-income countries, with a stillbirth rate beyond 28 weeks of pregnancy that is 19.5% higher than that of the best-performing countries [1]. Stillbirth is a devastating pregnancy outcome with long-lasting social and emotional consequences for parents and families and wide-ranging economic impacts on health systems and society [2]. Stillbirth rates are often used globally as an important indicator of the quality of maternity care [3]. In Australia, the rate of late gestation stillbirth (at 28 weeks of gestation or beyond) has shown a small reduction over the past two decades, from 3.3 per 1,000 births in 2003 to 2.5 per 1,000 births in 2021, and more work is required [4, 5]. It is estimated that up to 30% of late gestation stillbirths are potentially preventable, with closer attention to risk factors and better antenatal care [6, 7]. In response to the slow reduction in stillbirth rates, the Safer Baby Bundle (SBB) [8] was developed in 2018 and commenced implementation nationally. The SBB addresses commonly identified evidence practice gaps across five antenatal care components: 1) supporting women to stop smoking during pregnancy, 2) improving detection and management of fetal growth restriction (FGR), 3) raising awareness and improving care for women with decreased fetal movements (DFM), 4) improving awareness of maternal safe going-to-sleep position in late pregnancy, and 5) improving decision making about the timing of birth for women with risk factors for stillbirth [8].

The steps taken to review existing evidence and practice and gain expert consensus and endorsement for SBB recommendations are outlined elsewhere and include systematic evidence reviews, stakeholder consultation, and endorsement by national professional bodies [9]. Each of the five elements is underpinned by an evidence summary and, in the case of DFM, a national clinical guideline [10–14]. The recommendations were first released and endorsed in 2019 and last updated in 2023 to reflect emerging evidence and best practice.

Jurisdictions within Australia partnered with the Centre of Research Excellence in Stillbirth (Stillbirth CRE) to implement the SBB [9]. State-led quality improvement (QI) programs oversaw the implementation of the SBB. The aim of this paper is to describe a novel approach to implementation of an evidence-based bundle of antenatal care in Queensland. Using a modified Breakthrough Series (BTS) Collaborative approach, it was considered improvement was achievable at scale despite omitting four BTS core elements, thought to add to implementation burden on clinicians. The Safer Baby Improvement Project (SBBIP) was a statewide improvement project that aimed to reduce stillbirth rates by improving compliance with bundle elements. Challenges included COVID-19 having just emerged in Queensland as part of the global pandemic, resulting in lockdowns, isolation and restrictions on face‒to-face interactions that impacted the project. The impact on the maternity care workforce was significant, with furloughed staff reducing the number of clinicians available for front-line duties. A less burdensome approach to improvement was needed, leveraging the successful BTS.

Although observing a reduction in stillbirth rates within the short implementation timeframe was an ambitious aim, early evidence from Victoria indicates that SBB implementation was associated with a reduction in perinatal mortality without increasing iatrogenic or neonatal harm [15]. This suggests that the bundle approach has potential to improve outcomes, while highlighting the need for ongoing research to determine which interventions most effectively reduce stillbirth.

## Methods

This study used a before-and-after multimethod design to evaluate the program. Routinely collected perinatal data, clinical audits (14,800), project administrative data, and surveys of healthcare professionals (HCPs) and women receiving antenatal care (1,100) were used to measure improvements before and after implementation, and logistic regression analyses were used for comparisons of the outcomes. Qualitative feedback was obtained from learning session evaluation responses and from feedback during coaching calls. This was analysed using a pragmatic content analysis approach, where two project team members reviewed and summarised recurring ideas and themes to assess the perceived usefulness of the improvement approach. This study design complies with the StaRI standard for reporting implementation studies [16].

### Strategy

**Fig. 1:**
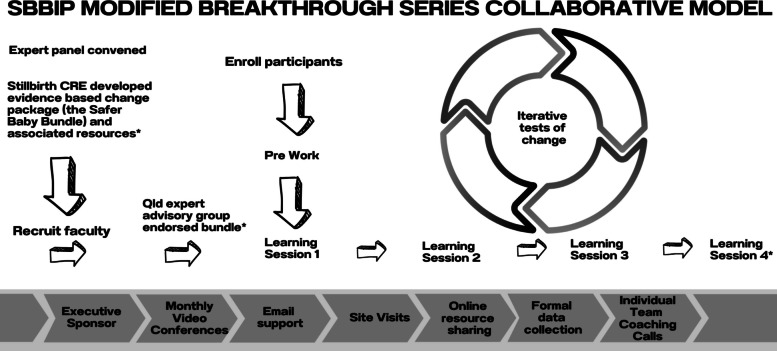
Modified Breakthrough Series Collaborative Model used for SBBIP. *Indicates methodology that was modified from the original Breakthrough Series Collaborative Model [17]

A modified BTS Collaborative approach [17] was used to deliver the SBBIP over other implementation strategies, as it is widely used to deliver improvement in health care settings around the world, and its ability to effect rapid change at scale, to close the gap between known science and daily practice. The Institute for Healthcare Improvement developed the BTS in 1991 to help health care organisations make "breakthrough" improvements in quality while reducing costs. A BTS is a short-term (6 to 15 month) learning system that brings together a large number of teams from hospitals or clinics to seek improvement in a focused topic area [17]. The methodology is prescriptive and described in *The Breakthrough Series white paper* [17]. Figure 1 describes the SBBIP-modified approach.

The project employed the same core components as prescribed by the original BTS model, such as a philosophy of ‘all teach – all learn’ [18], involving a community of practice facilitating regular meetings using structured agendas, measurement of progress against key performance indicators, and open sharing of ideas for improvement, supported by expertise in improvement and implementation science.

The modified BTS approach followed most of the prescribed elements described in previous studies, the Innovation Series [18] and the BTS white paper [17]. The SBBIP omitted the use of four BTS elements, as the project team’s experience found that clinicians placed limited value on these in achieving their goals. The SBBIP approach excluded routine testing of ideas via the plan-do-study-act (PDSA) cycle, instead relying on peer feedback. In place of Shewhart charts (statistical process control charts – SPC charts) to monitor performance in near real time [17, 19], a clinical quality improvement dashboard was developed. This dashboard made previously unavailable data accessible to frontline teams with minimal reporting burden. Training in the theory of improvement science was limited when compared to a traditional Collaborative [20]. There was also no requirement for regular formal reporting or monthly project progress scoring, which is commonly used as part of the BTS approach. Sites were asked to develop storyboards to periodically document their improvement journey, and a self-assessment was completed at the end of the project, documenting what was implemented.

### Project setting and training

Hospital and Health Services (HHSs) aimed to establish a project team composed of a ‘travel team’ of fewer than five people to attend learning sessions and a broader home team to progress the implementation of change ideas. Teams who were actively engaged in the project were defined as those that attended at least one learning session and submitted audits and/or women's and HCPs’ surveys. SBB eLearning completions also served as a measure of engagement.

All HHS teams were requested to complete the SBB eLearning module developed by the Stillbirth CRE [21] prior to the commencement of the project. Teams allocated a project lead (‘site champion’) to coordinate their local improvement efforts and liaise with the central CEQ project lead. The Stillbirth CRE provided national resources on each of the bundle components for healthcare professionals and women and their families (including factsheets, flyers and videos) via their website. Training was provided to project leads on the use of the central database to enter data from chart audits. QR codes were developed and made available for sites to easily access surveys of HCPs and women.

Three two-day learning sessions were held, two of which were in person (the first was virtual due to COVID-19). While it is not uncommon for additional learning sessions to be added for longer programs [16], a final one-day showcase (learning session) was included to close out the program, share successes and report final outcomes.

At each learning session, teams presented their project progress via story boards to their peers. Outside of the learning sessions, networking occurred on optional monthly videoconference calls and via a Microsoft Teams channel*.* Coaching calls with each team were scheduled monthly for 1:1 assistance, and some sites also received site visits prompted by requests.

### Measurement strategy

Teams were provided with a measurement and data collection plan including indicators from a family of measures, access to a central database to enter their local audit data, and a performance dashboard to display progress, which was centrally populated with system-level data where available. A family of measures include performance measures from three categories (outcome, process and balancing measures), to get a complete picture of changes in a complex system, ensuring improvements in one area do not harm others and that the core goal is met. Local teams were requested to complete audits based on whether they were birthing sites or non-birthing and how many births their service performed each year (see Table 1 below). Sample size calculations were based on the practicality of manual data collection.

**Table 1:** Sample size for SBBIP chart audits (birthing and non-birthing sites)

Birthing Sites	
With > 1000 births per year	Audit minimum of 60 women per calendar month
With > 500 and < 1000 births per year	Audit minimum of 30 women per calendar month
< 500 births per year	Audit minimum of 15 women (or all women if less than 15) per calendar month
Non-Birthing Sites	
Non-birthing sites	Audit minimum of 15 women (or all women if less than 15) per calendar month

Survey instruments (women and HCP surveys) were also provided for teams to administer. An electronic survey link was given to the women postnatally prior to discharge. The purpose of the survey was to better understand women’s experience of care practices relevant to each of the SBB components. These surveys were collected prior to SBBIP implementation and for the duration of the project (see Table 4 for a further breakdown of survey numbers received before and after implementation).

HCP surveys conducted by the Stillbirth CRE before and after implementation are reported elsewhere [22]. This survey sought to explore HCP practices in relation to stillbirth risk reduction education and SBB element application in everyday practice.

### Measures

The sharing of information and performance data is essential for driving service quality improvement [23]. An overarching national set of recommended SBB outcome, process, and balance measures was proposed through consultation with national stakeholders [9, 24]. For the SBBIP, these measures were further refined by a dedicated measurement working group comprising CEQ data analysts and leading HCPs. Additionally, local service quality improvement teams identified measures relevant to their improvement goals. A benchmarking system incorporating targets was integrated into the performance dashboard, facilitating improvement efforts and enabling sites to compare their outcomes against the state averages and peer sites. Table 2 below contains the list of measures used (excluding optional additional measures), and more detail is provided in Additional file 1.

**Table 2:** List of measures used for the Safer Baby Bundle Improvement Project for singleton births

Measure type	Measure
Outcome	Rate of stillbirths at 28 weeks or more gestation, excluding lethal congenital abnormalities in singleton births
Process	^*^Percentage of women who cease smoking between the first antenatal visit and birth
	^*^Percentage of women, identified as smoking, who were provided smoking cessation advice
	Proportion of term births with undetected FGR defined as severely growth restricted singletons (less than 3rd centile) undelivered at 40 weeks’ gestation (missed FGR)
	Proportion of babies delivered for suspected FGR at 37 weeks’ gestation or more who have a birthweight > 25th centile (false positive FGR)
	Percentage of women with documented risk assessment for FGR at first antenatal/booking visit
	Percentage of women with symphyseal fundal height (SFH) measurement taken and plotted on growth chart from 24 weeks gestation
	Proportion of women (at any gestation) identified as at risk of FGR whose care was escalated as per the FGR care pathway
	Percentage of women who attend for a Cardiotocograph—CTG (or Doppler) within 2 h of presentation with decreased fetal movement (DFM), from 28 weeks’ gestation
	^*^Proportion of women provided with maternal safe sleeping information by 28 weeks’ gestation
	^*^Proportion of women after 28 weeks’ gestation who report safe sleep practices (going-to-sleep on side)
	^*^Percentage of women who report being satisfied with their involvement in decision making around timing of birth
Balance	Rate of caesarean sections
	Percentage of inductions of labour or elective caesarean sections before 39 weeks with singleton pregnancy
	Percentage of babies admitted to Intensive Care Nursery (ICN) or Special Care Nursery (SCN) after 36 completed weeks
	Rate of late preterm births (gestation weeks 34–36 + 6) in singleton births

^*^includes multiple births

## Data collection, sources, time frame and reporting

### Clinical quality improvement dashboard

The dashboard was developed by the CEQ Analytics Team via Qlik Sense Software (Qlik Technologies Inc., Pennsylvania, United States). Clinical audit data were collected from patient medical records and entered into a secure online dataset via Research Electronic Data Capture (REDCap) [25, 26], which is hosted at CEQ, with direct export functionality into the dashboard. All other data were analysed in SAS Enterprise guide statistical package version 7.1 (SAS Institute Inc., Cary, NC, United States). The results, stored as patient-level data, were saved in Microsoft Excel files on an in-house virtual server at Queensland Health and subsequently uploaded into the dashboard. The patient’s hospital number and other identifying information were deidentified. Data were aggregated in Qlik Sense, to derive counts, rates and time trends for each measure from the study commencement to the most recent data update. Filters allowed stratification by pre- or post-implementation, facility, year, quarter and month. Dashboard access was provided to SBBIP, midwifery and obstetrics leaders at each site via a secure Qlik Sense portal. Data sources and outcomes for individual women were not linked. A full list of data sources is provided in Additional file 2.

#### Data collection time frame

**Fig. 2:**
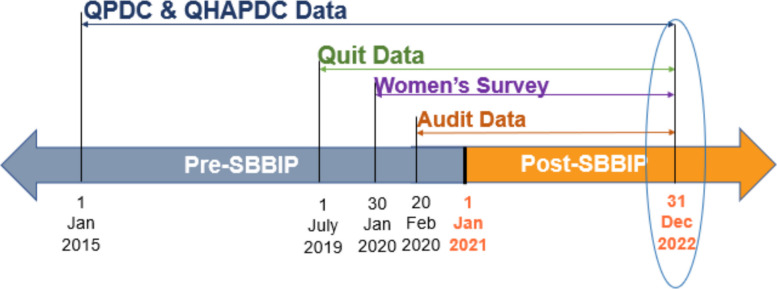
Time frame for each dataset used in the Safer Baby Bundle Improvement Project

SBBIP commenced on 01 January 2021. Data collection timelines and sources are shown in Fig. 2. Retrospective data for all measures based on the QPDC were collected from 01 January 2015, and Quit data were collected from 01 July 2019, both until the pre-SBBIP endpoint on 31 December 2020. For measures related to Women’s Survey, data collection commenced on 30 January 2020, and for the clinical audit, data collection commenced 20 February 2020, with both extending to the same endpoint. Post-SBBIP data were collected prospectively during implementation of the SBBIP until the end point on 31 December 2022.

### Outcomes

The key clinical outcome measure was a change from baseline in the rate of stillbirth post 28 weeks of gestation among singletons. The secondary measures included changes in process measures from baseline. Other outcomes included changes in balance measures (see Table 2).

The key project outcome was the number of sites that enrolled in the project that were actively engaged for the duration, implementing practice changes for one or more SBB components.

### Statistical analysis

The comparison of interest was the change in clinical practice before SBBIP implementation compared with that after SBBIP implementation. We performed logistic regression as the primary analytical approach for these comparisons for each measure. For measures based on the QPDC data, where sufficient data were available, we further adjusted the analyses for year (as a continuous variable) to account for temporal changes in the outcome of interest. The unadjusted and adjusted odds ratios (aORs) and 95% confidence intervals (CIs) are reported for each of the measures. Additional adjustments for nulliparity, age categories and BMI categories did not substantially alter the results. Therefore, the final model was adjusted solely for year.

We also conducted an interrupted time series (ITS) analysis for all measures derived from QPDC, given complete data availability across the project period. We analysed a single state-wide quarterly series using segmented Poisson regression with a log link, the log of the quarterly denominator as an offset and Pearson scaling for overdispersion, adjusting for seasonality by calendar quarter. We summarised effects as rate ratios (RR) with 95%CI. We assessed serial correlation using autocorrelation function plots and Ljung-Box tests of Pearson residuals and, where indicated, refitted models with an AR(1) residual correlation structure as a sensitivity analysis.

The study population for each measure varied according to the source of the data and definition of the measure. The main population of interest for this analysis was considered on the basis of the primary outcome: women with a singleton birth at or beyond 28 weeks of gestation, excluding termination of pregnancy and lethal congenital anomalies (Supplementary Material 1). The SBBIP was not powered to detect a difference in stillbirth rates before and after implementation. The demographic characteristics of the study population were compared between the pre-SBBIP and post-SBBIP phases using the Wilcoxon Two-Sample Test for continuous variables (equivalent to the Mann‒Whitney U test) and the X^2^ test for categorical variables.

All statistical tests were two-sided and we used p < 0.05 as the threshold for statistical significance. The data were analysed via SAS Enterprise guide statistical package version 7.1 (SAS Institute Inc., Cary, NC, United States).

### Ethics

This study was conducted with ethics approval obtained from the Royal Brisbane & Women’s Hospital Human Research Ethics Committee in June 2019 (approval number: HREC/2019/QRBW/47709).

## Results

### Program engagement

Forty-five antenatal services were eligible for enrolment. According to the SBBIP definition, 40 out of 45 enrolled teams were actively engaged in the project (89%), including 43 publicly funded hospitals with maternity services, one publicly funded private hospital and a non-birthing primary health centre, accounting for approximately 98% of births in public facilities in Queensland. While engagement was observed as a visible contribution to improvement efforts through attendance at learning sessions (Table 3) and story boards, contributing to data collection (Table 4), attendance at regular coaching calls and monthly all-in calls, and testing and implementing change ideas, not all engagement indicators were recorded.

**Table 3:** Attendance at learning sessions during SBBIP

Learning Session	Total n (%) of enrolled sites represented
Total number of attendees across all 4 learning sessions	395 (n/a)
Number of enrolled sites that were represented:	
1 Learning Session	45 (100%)
2 Learning Sessions	31 (69%)
3 Learning Sessions	29 (64%)
All 4 Learning Sessions	21 (47%)

**Table 4:** Surveys and audits undertaken during the SBBIP

Surveys and Audits	
Activity	Total n (%) of enrolled sites represented
Patient chart audits	14 800 (40%)
Women’s surveys (pre project Jan – July 2020)	356 (29%)
Women’s surveys (post project implementation Jan 2021—Dec 2022)	73 (57.9%)
HCP surveys (pre project)	573 (33%)
HCP surveys (post project)	204 (27%)

**Fig. 3:**
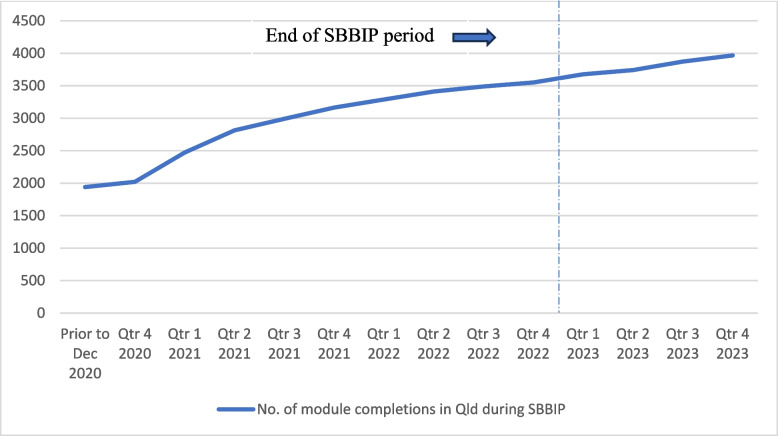
SBB e-learning module completion during SBBIP

While 66% of teams formally reported the implementation of one or more change ideas (through self-assessments or story boards), all sites (100%) are now using the Stillbirth CRE’s information resources for women and the statewide pregnancy handheld record, which incorporates SBB components into care. From October 2019–December 2023, 4000 clinicians in QLD completed the SBB e-Learning module. The number of completions continued to rise across the project period and after the project ended (Fig. 3). The SBB e-Learning modules were available before project commencement to provide background knowledge to the SBB bundle and its role in preventable stillbirth.

A subjective assessment of engagement was also made by the project team to include attendance at monthly coaching calls and statewide videoconferences, as these were not formally recorded. Executive sponsor engagement was variable. Subjectively, the project team reported that sites that had active executive sponsorship resulted in better engagement in the program, being more likely to attend coaching calls and learning sessions, implement change ideas and contribute to manual data collection.

An end of the project evaluation survey was conducted at the final learning session, where participants were asked to rate whether the support provided across the SBBIP was useful for their improvement efforts. The participants attending the learning session represented 26 different maternity services across QLD and included midwifery staff, obstetricians, consumer representatives and other key stakeholders (e.g., the Office of the Chief Nursing and Midwifery Office, Stillbirth CRE, Patient Safety and Quality). Among the 65 attending participants, 21 (32%) completed the survey, results of which are shown in Table 5.

**Table 5:** Usefulness of SBBIP support strategies

Strategies n = 21	Usefulness n (%)
Coaching calls	17 (80%)
Data collection tool (REDCap)	18 (86%)
Statewide dashboard	18 (86%)
Learning sessions	19 (90%)
Monthly statewide video conference calls	17 (80%)

Four learning sessions and three action periods were undertaken. Learning sessions were positively evaluated, with 312 of 395 (79%) rating them as very good or excellent. It was frequently reported in the evaluations that the highlights of the sessions were time made available to work with other team members and colleagues from other sites. Other reported themes included the opportunity to:

- Improve dialogue between HCPs and women on all components of the bundle.
- Enable more consistency in clinical practices and normalising stillbirth discussion with women.
- Have honest conversations around stillbirth with women.
- Unite efforts across the state on such an important topic.
- Standardise care, including resources available to women and staff.
- Consolidate information for women (i.e., brochures).
- Collaborate with peers.
- Improve general awareness of stillbirth through effective communication.

### Performance measures

There were demographic differences between the pre-SBBIP and post-SBBIP groups (Additional file 3: Table S1). Compared with pre-SBBIP women, post-SBBIP women were slightly older, with a median age of 30.0 years (IQR 26.0–33.0) versus 29.0 years (25.0–33.0) and a higher proportion aged 35 years or older (19.4% vs 17.2%). Post-SBBIP women also had a slightly greater median body mass index (BMI) of 25.3 kg/m^2^ (IQR 22.0–30.3) versus 24.5 kg/m^2^ (IQR 21.4–29.4) with a higher prevalence of obesity (26.0% vs 22.8%). The proportion of nulliparous women was also slightly greater post-SBBIP than pre-SBBIP (42.5% vs. 40.2%, Additional file 1).

### Process measures

#### Element 1: smoking cessation support

The rate of smoking cessation improved from 18.1% in the pre-SBBIP group to 24.3% in the post-SBBIP group (aOR 1.29 [95% CI 1.20–1.39]) (Table 6). No significant changes were observed between pre-SBBIP and post-SBBIP for other smoking cessation measures on the basis of audit data.

**Table 6:** Process measures

	**N**		**%**			
**Measure – source of data**	Pre-SBBIP	Post-SBBIP	Pre-SBBIP	Post-SBBIP	Effect size (95%CI): Post_SBBIP vs. Pre-SBBIP	Adjusted^a^ effect size (95%CI): Post-SBBIP vs. Pre-SBBIP
*Element 1: smoking cessation support*						
Quit smoking after 20 weeks of gestation – QPDC	7079/39185	3173/13078	18.1%	24.3%	**1.45 (1.39, 1.52)**	**1.29 (1.20, 1.39)**
Smoking cessation advice – Audit	229/299	1492/1905	76.6%	78.3%	1.10 (0.83, 1.47)	
Ceased smoking – Audit	82/299	492/1906	27.4%	25.8%	0.92 (0.70, 1.21)	
Quitline participation – Quit	450/1002	585/1417	44.9%	41.3%	0.86 (0.73, 1.02)	
Quitline completion – Quit	69/450	99/585	15.3%	16.9%	1.13 (0.80, 1.57)	
*Element 2: improved management of impaired fetal growth*						
Undetected severe FGR babies (missed FGR)—QPDC	1913/6366	540/2151	30.1%	25.1%	**0.78 (0.70, 0.87)**	1.11 (0.93, 1.31)
Planned births for suspected FGR (false positive FGR) in all singleton births at ≥ 37 weeks – QPDC	1584/239045	566/84976	0.66%	0.67%	1.01 (0.91, 1.11)	**0.77 (0.67, 0.89)**
Documented risk assessment – Audit	834/2129	8817/13992	39.2%	63.0%	**2.65 (2.41, 2.91)**	
Escalation of care – Audit	688/2129	7655/13986	32.3%	54.7%	**2.53 (2.30, 2.79)**	
SFH measurement – Audit	285/2128	4761/13991	13.4%	34.0%	**3.34 (2.93, 3.80)**	
*Element 3: improved management of decreased fetal movement (DFM)*						
DFM assessment within 2 h – Audit	592/618	3378/3473	95.8%	97.3%	**1.56 (1.00, 2.43)**	
DFM education – Women’s Survey	160/349	613/843	45.9%	72.7%	**3.15 (2.43, 4.08)**	
*Element 4: maternal safe sleeping advice*						
Safe sleeping education – Audit	219/2129	9464/13990	10.3%	67.7%	**18.24 (15.79, 21.07)**	
Safe sleeping practiced – Women’s Survey	113/349	658/843	32.4%	78.1%	**7.43 (5.63, 9.80)**	
*Element 5: improved decision-making on timing of birth for high-risk women*						
Satisfaction with decision making – Women’s Survey	287/349	741/843	82.2%	87.9%	**1.57 (1.11, 2.21)**	

*CI* confidence interval

effect estimate = odds ratio

^a^Effect size adjusted for year

Bold font indicates a significant change based on the 95% confidence intervals

#### Element 2: improved management of impaired fetal growth

The proportion of undetected severe FGR babies improved, decreasing from 30.1% in the pre-SBBIP period to 25.1% in the post-SBBIP period (Table 6).

The proportion of false-positive FGR babies among all singleton births at 37 weeks or beyond increased slightly from 0.66% pre-SBBIP to 0.67% post-SBBIP. However, after adjusting for temporal changes over the years in the adjusted model, the aOR was 0.78 [95% CI 0.70, 0.87), indicating a protective effect lower of SBB implementation.

Audit data demonstrated a significant improvement from post-implementation (Table 6). Documented risk assessment increased from 39.2% to 63.0% (OR 2.65 [95% CI 2.41–2.91]), appropriate escalation of care from 32.3% to 54.7% (OR 2.53 [95% CI 2.30–2.79]), and SFH measurement from 13.4% to 34.0% (OR 3.34 [95% CI 2.93–3.80]).

The ITS findings for QPDC-driven process measures were broadly consistent with the primary analyses. Changes were mainly seen in trends over time, with an immediate step change for quit smoking. Where AR(1) fitted, estimates were similar but less precise and did not change the overall interpretation (Additional file 4: Tables S2 and S3).

#### Element 3: improved management of decreased fetal movement (DFM)

Noting the high baseline compliance, there was still a modest increase pre- to post-SBBIP for DFM assessment within two hours from 95.8% to 97.3% (OR 1.56 [95% CI 1.00–2.43]) before to after SBBIP for the DFM assessment. Additionally, the proportion of women reporting they received education about DFM improved from 45.9% to 72.7% (OR 3.15 [95% 2.43–4.08]).

#### Element 4: maternal safe sleeping advice

Maternal safe side sleeping education improved from 10.3% pre-SBBIP to 67.7% post-SBBIP (OR 18.24 [95% CI 15.79–21.07]), and the proportion of women who reported safe going-to-sleep on side sleeping practices increased from 32.4% to 78.1% (OR 7.43 [95% CI 5.63–9.80]).

#### Element 5: improved decision-making on the timing of birth for high-risk women

The percentage of women reporting satisfaction with their involvement in decision-making regarding the timing of birth improved from 82.2% pre-SBBIP to 87.9% post-SBBIP, with an OR of 1.57 [95% CI 1.11–2.21]. See Table 6.

### Balance measures

**Fig. 4:**
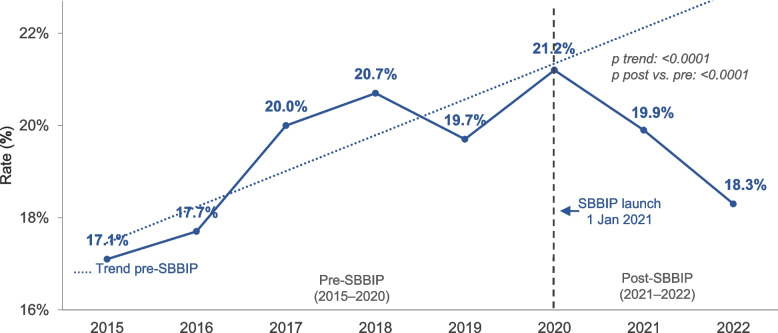
Trend in induction of labour or caesarean section before 39 weeks. Vertical line marks SBBIP commencement (1 Jan 2021). Points show annual rates for each calendar year. P-value for trend from Wald test based on parameter estimate and SE from logistic regression. P value for comparison between post-SBBIP and pre-SBBIP from the Wald test on the basis of the parameter estimate and SE from logistic regression

The overall rate of induction of labour or elective caesarean sections before 39 weeks decreased from 19.5% pre-SBBIP to 19.1% post-SBBIP (Table 2, Fig. 4). The adjusted model revealed a more pronounced effect of the SBBIP, significantly reducing the odds of these interventions (aOR 0.82 [95% CI: 0.80–0.84]).

The proportion of babies admitted to the neonatal intensive care unit (NICU)/special care nursery (SCN) after 37 weeks increased from 13.9% before implementation to 14.8% after implementation (Table 7). However, the adjusted model revealed a statistically significant reduction in the odds of admissions of these babies to NICU/SCN (aOR 0.91 [95% CI: 0.88–0.94]).

**Table 7:** Balance measures

	**N**		**%**			
Measure^*^	Pre-SBBIP	Post-SBBIP	Pre-SBBIP	Post-SBBIP	Effect size (95%CI): Post-SBBIP vs.Pre-SBBIP	Adjusted^a^ effect size (95%CI): Post-SBBIP vs. Pre-SBBIP
Caesarean sections in singleton pregnancy	76,534/258747	30,122/91671	29.6%	32.9%	**1.17 (1.15, 1.18)**	1.01 (0.99, 1.04)
Inductions of labour or elective caesarean sections before 39 weeks in singleton pregnancy	50,358/258747	17,518/91671	19.5%	19.1%	**0.98 (0.96, 1.00)**	**0.82 (0.80, 0.84)**
Babies admitted to Intensive Care Nursery or Special Care Nursery after 36 completed weeks in singleton pregnancy	35,755/256854	13,435/90883	13.9%	14.8%	**1.07 (1.05, 1.1)**	**0.91 (0.88, 0.94)**
Late preterm singleton births	13,330/258747	4462/91671	5.2%	4.9%	**0.94 (0.91, 0.98)**	0.98 (0.93, 1.03)
Early-term singleton births	76,935/258747	27,871/91671	29.7%	30.4%	**1.03 (1.02, 1.05)**	**0.86 (0.83, 0.88)**

*CI* confidence interval

effect estimate = odds ratio

^a^Effect size adjusted for year

^*^Source of data: QPDC

Bold font indicates a significant change based on the 95% confidence intervals

**Fig. 5:**
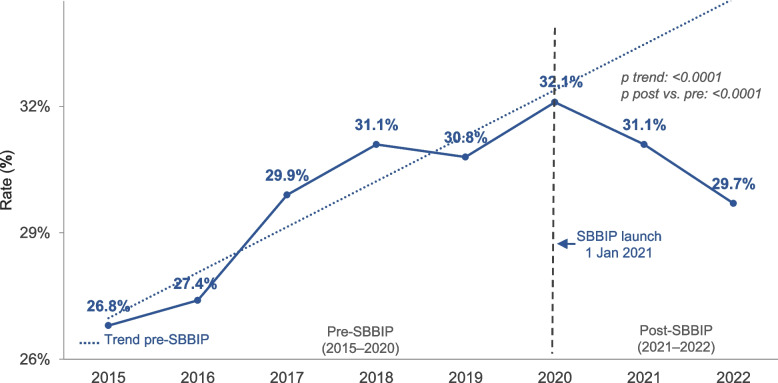
Trend in early-term singleton births. Early-term birth is defined as births at 37–38 + 6 weeks’ gestation. Vertical line marks SBBIP start (1 Jan 2021). Points show annual rates for each calendar year. P-value for trend from the Wald test based on parameter estimate and SE from logistic regression. P value for comparison between post-SBBIP and pre-SBBIP from the Wald test on the basis of the parameter estimate and SE from logistic regression

The rate of early-term births increased slightly from 29.7% to 30.4% after SBBIP (Table 7). After adjusting for year, the odds of early-term births in the post-implementation period significantly decreased, with an OR of 0.86 [95% CI 0.83–0.88] (Table 7, Fig. 5).

**Fig. 6:**
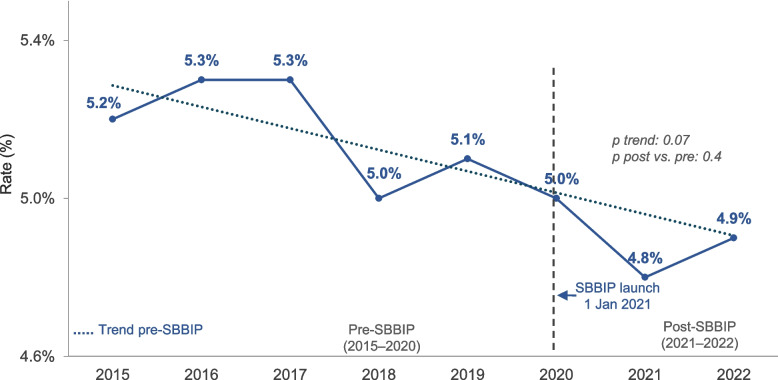
Trend in late preterm singleton births. Late preterm birth is defined as births at 34–36 + 6 weeks’ gestation. Vertical line marks SBBIP start (1 Jan 2021). Points show annual rates for each calendar year. P value for trend from Wald test based on parameter estimate and SE from logistic regression. P value for comparison between post-SBBIP and pre-SBBIP from Wald test on the basis of the parameter estimate and SE from logistic regression

There was no statistically significant change in the rate of caesarean sections and late preterm births (Fig. 6) from pre-SBBIP to post-SBBIP (Table 7).

The ITS findings for balance measures were consistent with the adjusted OR results, with changes mainly reflected as shifts in trend rather than abrupt level changes. AR(1) sensitivity analyses did not materially alter overall interpretation where applied (Additional file 4: Tables S2 and S3).

### Outcome measures

The rate of stillbirth at 28 weeks or beyond remained unchanged: 2.10 per 1000 births pre-SBBIP and 2.15 per 1000 births post-SBBIP, with an aOR of 1.24 [95% CI 0.96–1.60]. ITS findings were consistent, with no evidence of an implementation-related level change or change in trend, despite a small pre-SBBIP downward trend (Additional file 4: Table S2).

## Discussion

The SBBIP implemented an evidence-based bundle of care to reduce the risk of stillbirth in Queensland. The process indicators demonstrate the project was successful in delivering improvements in all bundle indicators thought to influence stillbirth rates. Stillbirth rates did not improve post-implementation, and while point estimates suggested a small increase, the evidence was inconclusive. It is possible that, while the observed improvements in care quality and consistency were important for reducing variation and enhancing best practice, they did not translate into a clinical change sufficient to impact overall stillbirth reduction.

The SBBIP methodology borrowed heavily from the traditional BTS Collaborative, however, was modified for health services challenged by unprecedented workload, staffing shortages, a global pandemic, record migration rates feeding a population boom in Queensland, and a small CEQ project team. It was hypothesised that removing some elements of a BTS would reduce the burden of improvement and have a limited overall impact on the results, as it would allow local teams to focus more on implementation and less time on documentation and formal tests of change.

BTSs are known to be resource intensive, requiring sustained effort over 12–18 months and physical time away from the clinical area [27]. It was not known if the removal of these elements would negatively impact the overall results. The project did not set out to measure the impact of removing these elements. Rather, determine if the approach would still result in an improvement in adoption of the bundle components, which it seemed to. It was also not mandatory to adopt all bundle components at each site and the SBB elements were not weighted in terms of their impact on stillbirth reduction. As there was no comparative group, we were not able to determine if the BTS elements removed resulted in the project being more or less effective. However, the changes did result in an improvement, suggesting removal of the four BTS elements did not adversely impact the outcome and that the modified BTS methodology was successful.

Care bundles offer a structured, evidence-based approach that reliably improves processes of care and patient outcomes, particularly when implemented using multifaceted strategies such as audit and feedback [28–30].

**Fig. 7:**
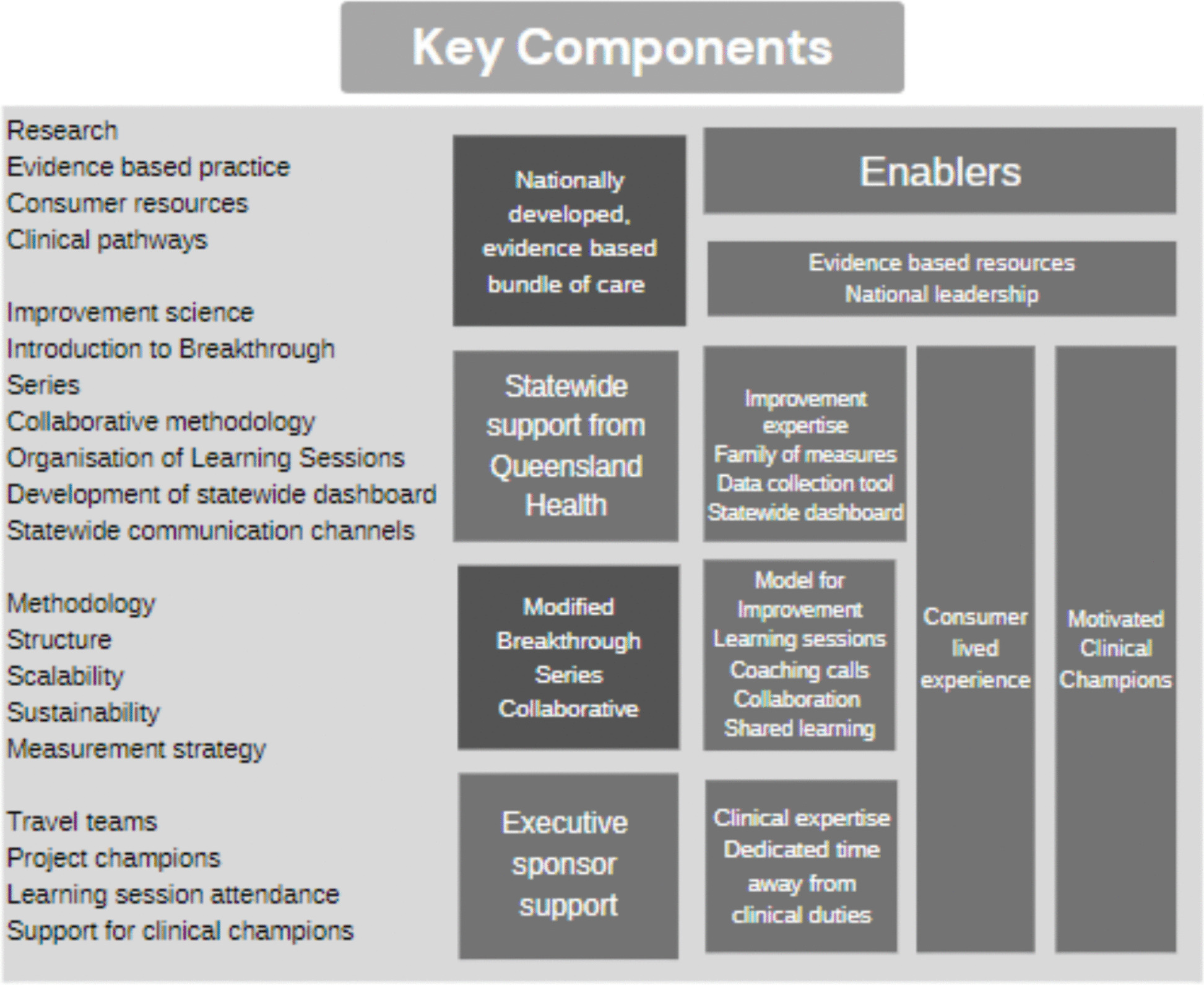
Modified BTS approach

There is limited information on the attributes of a BTS most likely to facilitate change [31]. We therefore did not know which elements were essential to affect a positive outcome. The CEQ project team decided to exclude the BTS elements from the SBB improvement approach we thought would least likely impact the overall outcome. The hypothesis was that improvements in clinical practice could still be achieved without the use of formal PDSA cycles, monthly reports, project progress score updates and SPC charts if all the other core elements were maintained (Fig. 7). The team was conscious of not removing too many essential building blocks and risking success of the project [32].

Reporting quality performance data as a family of measures with identified benchmark targets can guide improved practice in individual facilities [33]. Quality dashboards can support service level improvement by displaying data for quick retrieval, identifying trends over time, and importantly, facilitating detailed comparisons of outcomes across services [23, 34]. The CEQ central project lead monitored the SBBIP dashboard, using run charts that were discussed with teams at coaching calls. Plotting data over time via a run chart is a simple and effective way to determine whether the changes you are making are leading to improvement [17, 35]. Noting that sites rarely self-initiated access to the dashboard, a model where reports from the dashboard are provided to teams via a ‘push’ system to inform progress might be more helpful than a ‘pull’ system where teams are encouraged to access their data independently. Notably, rural sites were unable to download the dashboard easily because of digital bandwidth issues and reported they were unlikely to access it independently. Whereas traditional BTS collaboratives use data to drive improvement, data were used in the SBBIP to evaluate the impact, as the data was always retrospective.

Additionally, a learn-by-doing approach was used, which involved limiting the theory of improvement science compared to a traditional Collaborative approach [20]. Improvement science theory was provided at learning sessions; however, it was not a major focus or reinforced in statewide coaching calls. This was an attempt to reduce the burden on teams to comply with the improvement effort. Teams were encouraged to develop and agree on ideas to embed SBB elements into ‘business as usual’ and seek peer feedback, without additional documentation requirements; however, the lack of documentation made assessment by the CEQ project team difficult.

Teams were afforded the opportunity to be part of a national movement, providing a well-resourced package ready for implementation. Teams learned they were part of a larger cause and came together from across Queensland to learn about evidence and improvement science, to share successful strategies across sites, and to work with their local teams to plan their delivery. The cultural change achieved may be more important than the specific Collaborative elements used [27].

The SBBIP did not achieve a reduction in stillbirth rates in Queensland within the life of the project; however, the stillbirth rate also did not increase during that period, which coincided with the global COVID-19 pandemic. We analysed births to the end of 2022, aligned with the SBBIP evaluation window. Stillbirth at 28 weeks’ gestation or beyond is rare, and a post hoc sample size calculation indicated that detecting a 20% reduction with 80% power (two-sided alpha 0.05) would require about 117,701 post-SBBIP births, compared with 90,724 births available in this analysis. Making any improvements at scale takes time, usually longer than the project allows for a shift in outcome measures, so to determine if improvement has truly occurred and is sustained, it is necessary to observe patterns over time.

## Limitations

There has not been enough time post-implementation to observe the outcome results. The post-SBBIP sample size may have limited power to detect modest changes in stillbirth rates. Importantly, the published National Safer Baby Bundle study protocol [36, 37] specifies a predefined analysis endpoint (data collection to end December 2023) to measure the primary outcome of change in stillbirth rates at 28 weeks' gestation or more across Australian maternity settings before and after SBB implementation. Improvements are anticipated once all components of the SBB are fully embedded in standard care and practices.

Surveys were given to the women postnatally prior to hospital discharge. The numbers of surveys before and after were significantly different, with very small numbers post-survey compared to pre-survey. It is hypothesised the length of the survey may have contributed to the difference, and there may have been bias toward fewer respondents who were more engaged and more likely to be positive. Recall of information received by women is also subjective.

Participation in the surveys and clinical audits was also lower than planned. This may limit representativeness and introduce selection bias, so these findings should be interpreted cautiously.

The dashboard was only able to provide an indication of progress over time and was not linked with specific change ideas. Real-time dashboards are encouraged to better link the improvement effort with change effort performance. The project dashboard used lag data, sometimes more than three months behind, to report outcomes.

As there was no control group, comments cannot be made as to whether there could have been a larger improvement in process measures and a positive shift in the outcome measure if all BTS Collaborative elements were included. As SBB elements are not routinely collected, it is unclear if the improvements were sustained beyond the project.

## Conclusion

A modified BTS approach is a useful improvement model for implementing meaningful improvements in clinical practice. While it is unclear how many core BTS elements could be removed from a traditional approach as prescribed by the IHI, it appears that by maintaining the majority, minus four elements (documenting PDSA cycles, use of SPC charts, monthly reporting, and monitoring project progress scores), improvement at scale was still realised.

## Supplementary Information

Additional file 1: Word docx; SBBIP Measurement Strategy; Summary of process, outcome and balancing measures, goals, numerators and denominators, operational definitions.

Additional file 2: Word docx; Data Sources; where data was derived.

Additional file 3: Word docx; Demographic Characteristics Table; limited list of demographic characteristics pre and post project comparison, including statistical significance.

Additional file 4: Tables S2-S3. Word docx; Table S2 presents interrupted time series model estimates for project measures. Table S3 presents interrupted time series model estimates for project measures with AR(1) residual correlation.

## Data Availability

The datasets generated and/or analysed during the current study are not publicly available owing to patient confidentiality and are available from the corresponding author upon reasonable request.

## Abbreviations

BMI: Body mass index
BTS: Breakthrough Series Collaborative
CEQ: Clinical Excellence Queensland
DFM: Decreased Fetal Movement
FGR: Fetal growth restriction
HCP: Healthcare professional
HHS: Hospital and Health Service
ICN: Intensive Care Nursery
IHI: Institute for Healthcare Improvement
PDSA: Plan‒Do‒Study‒Act
QHAPDC: Queensland Hospital Admitted Patient Data Collection
QPDC: Queensland Perinatal Data Collection
REDCap: Research Electronic Data Capture
SBB: Safer Baby Bundle
SBBIP: Safer Baby Bundle Improvement Project
SCN: Special Care Nursery
Stillbirth CRE: Centre of Research Excellence in Stillbirth
SPC: Statistical Process Chart
