# Supplementary material for: Implementing the Safer Baby Bundle for stillbirth prevention across Queensland maternity services using a modified breakthrough series collaborative
Source: Implement Sci Commun. 2026 Apr 13;7:98. doi: 10.1186/s43058-026-00921-2 (PMC13188634; doi:10.1186/s43058-026-00921-2)
Supplement: Supplementary file 1 — Additional file 1: Word docx; SBBIP Measurement Strategy; Summary of process, outcome and balancing measures, goals, numerators and denominators, operational definitions. [file 43058_2026_921_MOESM1_ESM.docx]

**Additional file 1**

**SBBIP Measurement Strategy**

**AIM**

The aim of the Safer Baby Bundle (SBB) Improvement Project is to reduce the rate of stillbirth of babies from 28 weeks’ gestation by 20% by 2023. This will be achieved by implementing the five Safer Baby Bundle components: smoking cessation support; improved detection and management of impaired fetal growth; increasing awareness and management of women with decreased fetal movements; provision of maternal safe sleeping advice; and improved decision-making around timing of birth for women with risk factors.

**OUTCOME MEASURE**

| Measure | Goal | Numerator / Denominator | Operational Definitions | Data Collection Guide |
| --- | --- | --- | --- | --- |
| **Rate of stillbirths at 28 weeks or more gestation, excluding lethal abnormalities.** | 20% reduction | **Numerator:** Number of stillbirths at 28 weeks or more gestation.  **Denominator:** All births* | **Stillbirth** is defined as birth without signs of life at 28 weeks or more.  Excluding termination of pregnancy and lethal congenital abnormalities:  Q00.0 Anencephaly  Q00.00 Anencephaly, unspecified Q00.01 Incomplete anencephaly Q00.02 Complete anencephaly Q00.03 Acrania  Q00.04 Acephaly  Q00.09 Other anencephaly Q00.1 Craniorachischisis Q60.1 Renal agenesis, bilateral Q60.6 Potter's syndrome  Q61.1 Polycystic kidney, autosomal recessive Q61.42 Cystic renal dysplasia, bilateral Q61.45 Renal dysplasia, bilateral  Q91.0 Trisomy 18, meiotic nondisjunction Q91.1 Trisomy 18, mosaicism  Q91.2 Trisomy 18, translocation  Q91.3 Edwards' syndrome, unspecified Q91.4 Trisomy 13, meiotic nondisjunction Q91.5 Trisomy 13, mosaicism  Q91.6 Trisomy 13, translocation  Q91.7 Patau's syndrome, unspecified | Perinatal Data Collection (PDC) |

**PROCESS MEASURES**

| **BUNDLE ELEMENT 1: Promoting smoking cessation at every episode of care** | | | | |
| --- | --- | --- | --- | --- |
| **Measure** | Goal | Numerator / Denominator | Operational Definitions | Data Collection Guide |
| **Percentage of women who cease smoking between the first antenatal visit and birth** | 30% | **Numerator:** The number of women who cease smoking between the first visit and birth  **Denominator:** The number of women identified as smoking | The total number of females who gave birth with a stated smoking status are included in the denominator.  Smoking refers to the use of cigarettes or inhaled tobacco. | Audit of pregnancy record and clinical record |
| **Percentage of women, identified as smoking, who were provided smoking** | 100% | **Numerator:** women who were provided smoking cessation advice.  **Denominator:** women identified as smoking. | Smoking cessation advice includes the offer or referral to a smoking cessation service. | Audit of pregnancy record |
| **cessation advice.** |  |  |  |  |
| **OPTIONAL:**  **Percentage of women who** | 100% | **Numerator:** number of women who undertake CO monitoring the first and a subsequent appointment | Recorded within the pregnancy record.  Tests surrounding this well part of the implementation. | Audit of pregnancy record / Survey of Women |
| **undertake carbon monoxide**  **(CO) breath analysis at the** |  | **Denominator:** all women who attend a booking appointment. |  |  |
| **first and a subsequent** |  |  |  |  |
| **appointment** |  |  |  |  |
| **Proportion of women**  **identified as smokers who are referred to QFYQFB** | 50% | **Numerator:** number of women who are referred  **Denominator:** number of women identified as smoking | Quit for you, Quit for Baby program (QFYQFB) Quitline program.  Excludes referrals to other smoking cessation services. | Numerator - collected by Quitline  Denominator – Audit of pregnancy record |
| **program** |  |  |  |  |
| **No. women who participate in QFYQFB (Complete call 1)** | 50% | **Numerator:** number of women who participate (complete call 1)  **Denominator:** number of women referred | Participation is the completion of a single call from Quitline | Collected by Quitline |

| **No. participants who complete the QFYQFB program (Complete 4 calls)** | 20% | **Numerator:** number of women who complete the program (complete 4 calls)  **Denominator:** number of women who participate (complete call 1) | Completion is women who participate in all four calls for the program. | Collected by Quitline |
| --- | --- | --- | --- | --- |
| **Quit rates for participants who complete the QFYQFB program** | 30% | **Numerator:** number of women who have quit smoking six months post program completion.  **Denominator:** number of women who complete the program (complete 4 calls) | Women who continue with cessation at six months post completion of the QFYQFB program. | Collected by Quitline |
|  |  |  |  |  |
| **BUNDLE ELEMENT 2: Detection and management of fetal growth restriction** | | | | |
| **Measure** | Goal | Numerator / Denominator | Operational Definitions | Data Collection Guide |
| **Proportion of term births with undetected FGR defined as severely growth restricted singletons (less than 3rd centile) undelivered at 40**  **weeks’ gestation (missed**  **FGR*)** | Nil | **Numerator:** Singleton births at 39 completed weeks or more gestation with birthweight less than 3rd centile according to Dobbins et al  **Denominator:** Singleton births (live and stillborn) with severe FGR born at 32 weeks or more gestation | *This indicator shows the proportion of severely growth restricted singleton babies (defined as birth weight below the third centile, corrected for gestational age, plurality and sex) who were born at or after 40 weeks’ gestation. Excluding termination of pregnancy and lethal congenital abnormality.  **39 completed weeks is equal to 40+0 weeks** | Perinatal Data Collection (PDC) |
| **Proportion of babies delivered for suspected FGR at 37 weeks’ gestation or more who have a birthweight**  **>25th centile.** | Nil | **Numerator:** Number of singleton babies delivered at 37 weeks’ gestation or more for suspected FGR who have a birthweight >25th centile.  **Denominator:** Number of singleton births at 37 weeks’ gestation or more | This measure is detecting of the babies who were delivered by induction of labour or caesarian section for FGR and how many were NOT FGR.  Excluding termination of pregnancy and lethal congenital abnormality. | Perinatal Data Collection (PDC) |
| **Percentage of women with documented risk assessment for FGR at first antenatal / booking visit.** | 80% | **Numerator:** Women with documented risk assessment for FGR at first antenatal booking visit  **Denominator:** All women attending for antenatal care. | Risk assessment must be documented within the pregnancy record. | Audit of pregnancy record |

| **Percentage of women with symphyseal fundal height (SFH) measurement taken and plotted on growth chart from 24 weeks gestation** | | 80% | **Numerator:** women with symphyseal fundal height (SFH) measurement taken and plotted on growth chart from 24 weeks gestation  **Denominator:** women having symphyseal fundal height (SFH) measurement | Assessment of fetal growth through serial measurement of symphysis-fundal height (SFH) is recommended at every antenatal appointment from 24 weeks gestation  A standardised approach to SFH measurement and serial plotting through use of standardised growth chart is recommended as part of this improvement project  Escalation of care should be considered when risk identified | | | Audit of pregnancy record |
| --- | --- | --- | --- | --- | --- | --- | --- |
| **Proportion of women (at any gestation) identified as at risk of FGR whose care was escalated as per the FGR care pathway** | | 100% | **Numerator:** Women (at any gestation) identified as at risk of FGR whose care was escalated as per FGR care pathway  **Denominator:** All women. | FGR, for the purposes of this project, is defined by the rate of growth according to serial fundal height or ultrasound EFW measurements (regardless of whether they are already below the tenth centile or not), with or without abnormal umbilical or fetal Doppler flow measurements.  Obstetric medical opinion for ongoing management when FGR is suspected.  The escalation of care must be documented within the pregnancy record. | | | Audit of pregnancy record |
|  | |  |  |  | | |  |
| **BUNDLE ELEMENT 3: Raising awareness and improving care for women with decreased fetal movements** | | | | | | | |
| **Measure** | | Goal | Numerator / Denominator | Operational Definitions | | | Data Collection Guide |
| **Percentage of women who attend for a CTG (or Doppler) within 2hrs of presentation with decreased fetal movement (DFM), from 28 weeks gestation** | | 100% | **Numerator:** women who attend for a CTG (or Doppler) within 2hrs after presenting with decreased fetal movement (DFM) from 28 weeks gestation  **Denominator:** women who have reported decreased fetal movement (DFM) from 28 weeks gestation. | Fetal movements have been defined as any discrete kick, flutter, swish or roll.  DFM is a maternal perception of a change to normal fetal movements.  DFM should be acted upon when a woman reports a reduction or sudden alteration in fetal movement.  Clinical management of women who report DFM is provided according to the PSANZ/Stillbirth CRE DFM guidelines  All women who report DFM in person or via phone should have the “DECREASED / CHANGED FETAL MOVEMENTS  RECORD” completed  All women from 28 weeks who have reported DFM are included in the denominator  All women who from 28 weeks who have reported DFM and attend for CTG within 2 hours are captured in the numerator.  Was DFM reported from 28 weeks of gestation?  If yes, was a CTG conducted in 2 hours after reporting? y/n  Time of presentation is identified in the antenatal assessment document or EDIS. | | | Audit of pregnancy record |
| **Proportion of women provided with DFM information by 28 weeks’ gestation.** | 100% | | **Numerator:** Women who were provided with DFM information by 28 weeks’ gestation.  **Denominator:** All women attending for antenatal care. | | Information provided may be in any format. | Survey of women | |
|  |  | |  | |  |  | |
| **BUNDLE ELEMENT 4: Improving awareness of maternal safe sleeping position** | | | | | | | |
| **Measure** | Goal | | Numerator / Denominator | | Operational Definitions | Data Collection Guide | |
| **Proportion of women provided with safe sleeping information by 28 weeks gestation** | 100% | | **Numerator:** Number of women who were provided with safe sleeping information by 28 weeks gestation.  **Denominator:** All women attending for antenatal care. | | Information provided consistent with the nation SBB safe sleeping position material. | Audit of pregnancy record | |
| **Proportion of women after 28 weeks’ gestation who report safe sleep practices (side sleeping).** | 90% | | **Numerator:** Number of women after 28 weeks’ gestation who can describe safe late-pregnancy sleep practices (going to sleep on their side).  **Denominator:** Number of women attending for antenatal care after 28 weeks’ gestation. | | Safe sleeping is settling to sleep on either side. | Survey of women | |
|  |  | |  | |  |  | |
| **BUNDLE ELEMENT 5:** |  | |  | |  |  | |
| **Measure** | Goal | | Numerator / Denominator | | Operational Definitions | Data Collection Guide | |
| **Percentage of women who report being satisfied with their involvement in decision making around timing of birth** | 80% | | **Numerator:** all women who report being satisfied with their involvement in decision-making around timing of birth  **Denominator:** All women at birth | | Shared decision making involves the integration of a patient’s values, goals and concerns with the best available evidence about benefits, risks and uncertainties of treatment, to achieve appropriate health care decisions.  When planning the birth of a baby with suspected FGR the aim is achieve the maximum maturity possible where it is safe to do so. Benefits of early birth to reduce stillbirth need to be carefully weighed against the risk of intervention for the baby at a given gestation. Care should be individualised and woman-centered, using decision aids where possible. The following points should be considered and discussed:   - Woman/family preferences - Maternal conditions - Gestational age, EFW and fetal condition - Intrapartum monitoring - Access to appropriate neonatal services | Survey of women | |

**BALANCE MEASURES**

| Measure | Goal | Numerator / Denominator | Operational Definitions | Data Collection Guide |
| --- | --- | --- | --- | --- |
| **Rate of caesarean sections** |  | **Numerator:** births by caesarean sections  **Denominator:** all births* | A surgical operation by which the baby is extracted through an incision in the abdominal and uterine walls. | Perinatal Data Collection (PDC) |
| **Percentage of inductions of labour or elective caesarean sections before 39 weeks with singleton pregnancy** |  | **Numerator:** women who have an IOL / elective caesarean section before 39 weeks gestation with singleton pregnancy  **Denominator:** all women who birth with singleton pregnancy | Use of intervention (medication, rupture of membranes, or mechanical means) to assist the process of labour to begin. | Perinatal Data Collection (PDC), Queensland Hospital Admitted Patient Data Collection (QHAPDC) & |
| **Percentage of babies admitted to Intensive Care Nursery (ICN) or Special Care Nursery (SCN) after 36 completed weeks** |  | **Numerator:** babies admitted to special care nursery after 36 completed weeks  **Denominator:** all live births. Excluding termination of pregnancy | All babies that have an admission for, any reason, to special care nursery to be captured as part of the numerator. | Perinatal Data Collection (PDC) |
| **Rate of late preterm births** |  | **Numerator:** Number of all babies born between 34 and 36+6 weeks’ gestation  **Denominator:** all births | Preterm birth is defined as the three week period from 34 weeks gestation. | Perinatal Data Collection (PDC) |

**Definitions:**

***Birth:** A birth is defined as the event in which a baby comes out of the uterus after a pregnancy of at least 20 weeks gestation or weighing 400 grams or more
