# Supplementary material for: Implementing the Safer Baby Bundle for stillbirth prevention across Queensland maternity services using a modified breakthrough series collaborative
Source: Implement Sci Commun. 2026 Apr 13;7:98. doi: 10.1186/s43058-026-00921-2 (PMC13188634; doi:10.1186/s43058-026-00921-2)
Supplement: Supplementary file 2 — Additional file 2: Word docx; Data Sources; where data was derived. [file 43058_2026_921_MOESM2_ESM.docx]

Additional file 2

## Data sources

- The Queensland Perinatal Data Collection (QPDC) (1), providing routinely collected population-based surveillance system perinatal data covering all births in Queensland hospitals participating in the SBB. The scope of the collection includes all live births, and stillbirths of at least 20 weeks gestation and/or at least 400 grams in weight.
- The Queensland Hospital Admitted Patient Data Collection (QHAPDC)(2), collecting demographic data and clinical information on all admitted patients separated from both public and licensed private hospitals and private day surgeries in Queensland.
- Clinical audits and implementation process data collected from birthing and non-birthing sites participating in the SBB and providing antenatal care. This encompassed a retrospective audit of the pregnancy health record and/or clinical record using an audit tool specifically developed for the SBB improvement program in Queensland (and agreed upon by the National SBB Steering Committee). Each site is required to enter their audits monthly, with the number of audits requested based on their birth numbers. For sites with more than 1000 births per year, a minimum of 60 audits; sites with between 500 and 1000 births per year, a minimum of 30 audits; and for sites with less than 500 births per year, a minimum of 15 audits or all births if less than 15 births per month. Consecutive births are chosen to complete the audit to avoid selection bias.
- Surveys of women receiving antenatal care at participating sites. The survey is an abbreviated version of the National SBB pre-implementation survey of women(3, 4) . This is a brief self-administered on-line survey completed by women following the birth (before hospital discharge or within 6 weeks of birth). Women who had a stillbirth or neonatal death prior to discharge from the hospital of birth were excluded. Survey questions (n = 20) address demographic characteristics and explore women’s experience and views of care received around relevant SBB elements of care through a multiple-choice format. All women are offered to complete the survey in the post-natal period, prior to discharge. The minimum number of surveys for each site aligns with the numbers required for clinical audit based on birth numbers.
- Quitline data, specifically from the Queensland Health Quit for you, Quit for Baby program, a free quit smoking program for pregnant women and their partners. These data consist of the number of referrals from each site, the proportion of those women who accepted the telephone call to participate in the program, and the proportion on women referred who completed the program.
- Program administrative data- attendance at learning sessions, forum evaluations, site story boards, eLearning completion reports, agreements, coaching calls, changes to state forms.

1. Queensland Perinatal Data Collection (QPDC) Manual 2021-2022 [Internet]. 2021.

2. Queensland Hospital Admitted Patient Data Collection (QHAPDC) Manual 2021-2022 [Internet]. 2021.

3. Andrews CJ, Ellwood D, Middleton PF, Gordon A, Nicholl M, Homer CSE, et al. Implementation and evaluation of a quality improvement initiative to reduce late gestation stillbirths in Australia: Safer Baby Bundle study protocol. BMC Pregnancy and Childbirth. 2020;20(1):694.

4. Andrews C BF, Meredith N, et al. The Safer Baby Bundle: Pre- implementation surveys of women and maternity clinicians. In: Congress PSoAaNZ, editor. Perinatal Society of Australia and New Zealand Congress2021.
