# Supplementary material for: Implementing the Safer Baby Bundle for stillbirth prevention across Queensland maternity services using a modified breakthrough series collaborative
Source: Implement Sci Commun. 2026 Apr 13;7:98. doi: 10.1186/s43058-026-00921-2 (PMC13188634; doi:10.1186/s43058-026-00921-2)
Supplement: Supplementary file 3 — Additional file 3: Word docx; Demographic Characteristics Table; limited list of demographic characteristics pre and post project comparison, including statistical significance. [file 43058_2026_921_MOESM3_ESM.docx]

Additional file 3

**Table S1** Demographic characteristics

| **Characteristics**^a^ | **Pre-SBBIP**  **(N= 256250)** | **Post-SBBIP**  **(N= 90724)** | ***P*** |
| --- | --- | --- | --- |
| Age (years), Median (IQR) | 29.0 (25.0, 33.0) | 30.0 (26.0, 33.0) | < 0.0001 |
| Age (years), n (%) |  |  |  |
| <20 | 10909 (4.3%) | 2744 (3.0%) | < 0.0001 |
| 20-34 | 201265 (78.5%) | 70357 (77.6%) |  |
| ≥35 | 44076 (17.2%) | 17623 (19.4%) |  |
| Body mass index (BMI, kg/m²), Median (IQR)^b^ | 24.5 (21.4, 29.4) | 25.3 (22.0, 30.3) | < 0.0001 |
| BMI (kg/m²), n (%) |  |  |  |
| <18.5 (underweight) | 14338 (5.6%) | 3851 (4.2%) | < 0.0001 |
| 18.5 to <25 (normal) | 119664 (46.7%) | 39577 (43.6%) |  |
| 25 to <30 (overweight) | 59921 (23.4%) | 22805 (25.2%) |  |
| ≥30 (obese) | 58565 (22.8%) | 23614 (26.0%) |  |
| Unknown | 3762 (1.5%) | 877 (1.0%) |  |
| Nulliparous, n (%) | 102986 (40.2%) | 38532 (42.5%) | < 0.0001 |
| Previous stillbirth, n (%) | 3615 (1.4%) | 1351 (1.5%) | 0.09 |
| Baby’s gestational age at birth (weeks), Median (IQR) | 39 (38, 40) | 39 (38, 40) | < 0.0001 |

IQR: Interquartile range

^a^ Women with a singleton birth at ≥28 weeks of gestation, excluding termination of pregnancies and lethal congenital anomalies.

^b^ Excluded women with unknown BMIs (Pre-SBBIP: n= 3762; Post-SBBIP: n=877)

P-values, comparing Pre-SBBIP with Post-SBBIP, were generated using the Wilcoxon Two-Sample Test (equivalent to the Mann-Whitney U test) for continuous variables and the Chi-square (X^2^) test for categorical variables.
