# Supplementary material for: Implementing the Safer Baby Bundle for stillbirth prevention across Queensland maternity services using a modified breakthrough series collaborative
Source: Implement Sci Commun. 2026 Apr 13;7:98. doi: 10.1186/s43058-026-00921-2 (PMC13188634; doi:10.1186/s43058-026-00921-2)
Supplement: Supplementary file 4 — Additional file 4: Tables S2-S3. Word docx; Table S2 presents interrupted time series model estimates for project measures. Table S3 presents interrupted time series model estimates for project measures with AR(1) residual correlation. [file 43058_2026_921_MOESM4_ESM.docx]

Additional file 4

**Table S2** Interrupted time series model estimates for project measures

| **Measure** | **RR (95%CI)** | ***P*** |
| --- | --- | --- |
| ***Process measures*** |  |  |
| Quit smoking after 20 weeks of gestation |  |  |
| Level change at SBBIP implementation | 1.153 (1.056, 1.260) | 0.002 |
| Pre-SBBIP trend (per quarter) | 1.005 (1.002, 1.009) | 0.004 |
| Change in trend post vs pre | 1.020 (1.002, 1.037) | 0.028 |
| Post-SBBIP trend (per quarter) | 1.025 (1.008, 1.043) | 0.004 |
| Undetected severe FGR babies (missed FGR) |  |  |
| Level change at SBBIP implementation | 1.048 (0.889, 1.236) | 0.575 |
| Pre-SBBIP trend (per quarter) | 0.985 (0.979, 0.991) | <0.001 |
| Change in trend post vs pre | 1.006 (0.972, 1.041) | 0.729 |
| Post-SBBIP trend (per quarter) | 0.991 (0.958, 1.025) | 0.594 |
| Planned births for suspected FGR (false positive FGR) in     all singleton births at ≥37 weeks |  |  |
| Level change at SBBIP implementation | 0.890 (0.718, 1.104) | 0.29 |
| Pre-SBBIP trend (per quarter) | 1.019 (1.010, 1.028) | <0.001 |
| Change in trend post vs pre | 0.953 (0.911, 0.998) | 0.039 |
| Post-SBBIP trend (per quarter) | 0.971 (0.928, 1.016) | 0.201 |
| ***Balance measures*** |  |  |
| Caesarean sections in singleton pregnancy |  |  |
| Level change at SBBIP implementation | 1.011 (0.984, 1.039) | 0.437 |
| Pre-SBBIP trend (per quarter) | 1.007 (1.005, 1.008) | <0.001 |
| Change in trend post vs pre | 0.998 (0.993, 1.004) | 0.485 |
| Post-SBBIP trend (per quarter) | 1.005 (0.999, 1.010) | 0.105 |
| Inductions of labour or elective caesarean sections before 39 weeks in singleton pregnancy |  |  |
| Level change at SBBIP implementation | 0.941 (0.879, 1.007) | 0.078 |
| Pre-SBBIP trend (per quarter) | 1.010 (1.007, 1.013) | <0.001 |
| Change in trend post vs pre | 0.967 (0.954, 0.981) | <0.001 |
| Post-SBBIP trend (per quarter) | 0.977 (0.963, 0.991) | 0.001 |
| Babies admitted to Intensive Care Nursery or Special Care Nursery after 36 completed weeks in singleton pregnancy |  |  |
| Level change at SBBIP implementation | 0.954 (0.907, 1.002) | 0.062 |
| Pre-SBBIP trend (per quarter) | 1.010 (1.008, 1.012) | <0.001 |
| Change in trend post vs pre | 0.988 (0.978, 0.998) | 0.016 |
| Post-SBBIP trend (per quarter) | 0.997 (0.987, 1.007) | 0.583 |
| Late preterm singleton births |  |  |
| Level change at SBBIP implementation | 0.973 (0.912, 1.039) | 0.421 |
| Pre-SBBIP trend (per quarter) | 0.998 (0.995, 1.000) | 0.079 |
| Change in trend post vs pre | 1.002 (0.988, 1.015) | 0.81 |
| Post-SBBIP trend (per quarter) | 0.999 (0.986, 1.013) | 0.931 |
| Early-term singleton births |  |  |
| Level change at SBBIP implementation | 0.955 (0.917, 0.993) | 0.022 |
| Pre-SBBIP trend (per quarter) | 1.009 (1.008, 1.011) | <0.001 |
| Change in trend post vs pre | 0.979 (0.971, 0.987) | <0.001 |
| Post-SBBIP trend (per quarter) | 0.988 (0.980, 0.996) | 0.003 |
| ***Outcome measure*** |  |  |
| Stillbirth at ≥ 28 weeks |  |  |
| Level change at SBBIP implementation | 1.059 (0.755, 1.486) | 0.74 |
| Pre-SBBIP trend (per quarter) | 0.987 (0.974, 0.999) | 0.041 |
| Change in trend post vs pre | 1.053 (0.986, 1.125) | 0.124 |
| Post-SBBIP trend (per quarter) | 1.039 (0.974, 1.109) | 0.248 |

RR rate ratio

CI confidence intervals

We estimated RRs using segmented Poisson regression with a log link, an offset of log(denominator) and quarter as the time unit. Models adjusted for seasonality by including calendar quarter (Q1 to Q4) as a categorical covariate and used Pearson scaling to allow for overdispersion. We present RRs (95% CI) by exponentiating model coefficients. The four reported RRs correspond to the level change at SBBIP implementation, the pre-SBBIP trend per quarter, the change in trend post vs pre (SBBIP) and the post-SBBIP trend per quarter (pre-SBBIP trend plus change in trend). P values are two-sided and obtained from Wald tests for the reported RR estimates.

**Table S3** Interrupted time series estimates allowing AR(1) residual correlation

| **Measure** | **RR (95%CI)** | ***P*** |
| --- | --- | --- |
| ***Process measures*** |  |  |
| Quit smoking after 20 weeks of gestation |  |  |
| Level change at SBBIP implementation | 1.165 (1.035, 1.311) | 0.013 |
| Pre-SBBIP trend (per quarter) | 1.005 (0.998, 1.011) | 0.166 |
| Change in trend post vs pre | 1.018 (0.992, 1.045) | 0.161 |
| Post-SBBIP trend (per quarter) | 1.023 (0.999, 1.048) | 0.06 |
| Planned births for suspected FGR (false positive FGR) in     all singleton births at ≥37 weeks |  |  |
| Level change at SBBIP implementation | 0.916 (0.692, 1.212) | 0.525 |
| Pre-SBBIP trend (per quarter) | 1.019 (1.004, 1.034) | 0.012 |
| Change in trend post vs pre | 0.948 (0.890, 1.011) | 0.1 |
| Post-SBBIP trend (per quarter) | 0.966 (0.909, 1.027) | 0.257 |
| ***Balance measures*** |  |  |
| Inductions of labour or elective caesarean sections before 39 weeks in singleton pregnancy |  |  |
| Level change at SBBIP implementation | 0.948 (0.879, 1.023) | 0.158 |
| Pre-SBBIP trend (per quarter) | 1.012 (1.003, 1.021) | 0.011 |
| Change in trend post vs pre | 0.962 (0.936, 0.988) | 0.006 |
| Post-SBBIP trend (per quarter) | 0.973 (0.951, 0.996) | 0.025 |
| Early-term singleton births |  |  |
| Level change at SBBIP implementation | 0.964 (0.920, 1.011) | 0.129 |
| Pre-SBBIP trend (per quarter) | 1.009 (1.005, 1.013) | <0.001 |
| Change in trend post vs pre | 0.976 (0.963, 0.989) | 0.001 |
| Post-SBBIP trend (per quarter) | 0.985 (0.973, 0.997) | 0.017 |

RR rate ratio

CI confidence interval

We estimated RRs using a segmented Poisson model with a log link, an offset of log(denominator) and quarter as the time unit, allowing for first-order autoregressive correlation (AR(1)) in residuals across successive quarters. Models adjusted for seasonality by including calendar quarter (Q1 to Q4) as a categorical covariate. We present RRs (95% CI) by exponentiating model coefficients. The four reported RRs correspond to the level change at SBBIP implementation, the pre-SBBIP trend per quarter, the change in trend post vs pre (SBBIP) and the post-SBBIP trend per quarter (pre-SBBIP trend plus change in trend). P values are two-sided and obtained from Wald tests for the reported RR estimates.
